# Determinants of sexual health information-seeking on social media among Chinese college students: an application of the Comprehensive Model of Information Seeking

**DOI:** 10.3389/fpubh.2026.1874571

**Published:** 2026-09-01

**Authors:** Zhuowen Feng, Wei He, Jiaru Xie, Guancheng Wang, Wanfang Lin

**Affiliations:** 1 College of Literature and News Communication, Guangdong Ocean University, Zhanjiang, Guangdong, China; 2 College of Electronic and Information Engineering, Guangdong Ocean University, Zhanjiang, Guangdong, China

**Keywords:** Chinese college students, Comprehensive Model of Information Seeking, health behavior, new media, sexual health information seeking, social media

## Abstract

**Background:**

Chinese college students often use social media to access sexual health information in a context of uneven formal sex education and cultural sensitivity surrounding sexuality. Understanding the factors associated with this behavior can inform sexual health communication and education.

**Methods:**

A cross-sectional online survey was conducted with 283 Chinese college students. Structural equation modeling was used to examine the associations among perceived direct or vicarious experience with sexual health issues, risk-oriented salience, beliefs, perceived information carrier characteristics, perceived utility of sexual health information (SHI), and sexual health information-seeking behavior (SHISB).

**Results:**

Perceived direct or vicarious experience, risk-oriented salience, beliefs, and perceived information carrier characteristics were positively associated with the perceived utility of social media for SHI. Perceived information carrier characteristics and perceived utility of SHI were positively associated with SHISB. Perceived utility served as an indirect statistical pathway linking perceived information carrier characteristics and SHISB.

**Conclusion:**

The findings suggest that the Comprehensive Model of Information Seeking (CMIS) provides a useful but bounded framework for examining individual-cognitive and information-evaluation factors related to SHISB among Chinese college students. Improving the credibility, clarity, accessibility, and relevance of online sexual health information may better support students' sexual health information needs. Given the cross-sectional design and risk-oriented measurement, the findings should be interpreted as statistical associations rather than causal effects or a comprehensive account of sexual health practices.

## 1 Introduction

Sexual health, as defined by the World Health Organization (WHO), extends beyond the mere absence of illness, malfunction, or weakness.^1^ It encompasses a state of physical, emotional, mental, and social wellbeing in relation to sexuality. The pursuit of sexual and reproductive health information, along with the search for a partner, is deeply intertwined with everyday life, social adaptation, and the fulfillment of sexual curiosity. Research on the online seeking of sexual information falls within the broader domain of information behavior, exploring various facets of sexuality-related knowledge (1). According to Johnson and Meischke (2) information seeking can be defined as the intentional acquisition of relevant information from selected sources. Health information refers to the knowledge, technologies, skills, concepts, and behaviors related to health, including those transmitted and shared during health communication, and covers topics such as personal wellbeing, diseases, nutrition, and overall health (3). Bu et al. (4) asserted that health information-seeking behavior involves the use of the Internet to retrieve, browse, select, evaluate, and apply health-related knowledge to meet personal health needs. Strengthening sexual health education and intervention for key groups, particularly adolescents, is explicitly emphasized in the “Healthy China 2030" Plan. The healthy development of young people is critically dependent on their sexual health, which includes not only possessing adequate sexual awareness but also participating in healthy sexual practices (5). However, the 2019–2020 National College Sexual and Reproductive Health Survey Report highlights a significant gap, revealing that only 52% of college students have received formal sex education in the classroom (6). Zhang et al. (7) found that Chinese college students generally exhibit low levels of understanding regarding sexually transmitted diseases, human immunodeficiency virus/acquired immunodeficiency syndrome, condom use, reproductive health, and contraception. Furthermore, Li et al. (8) identified significant deficiencies in the content of sexual health education and a lack of skills in sexual health education for college students in Guangxi province, China. These findings underscore the ongoing gaps in the correct knowledge and understanding of sexual health among Chinese college students. However, sexual health information-seeking behavior (SHISB) among Chinese college students should not be understood only as a response to insufficient knowledge or formal sex education. Because sexuality is culturally and relationally sensitive, students' engagement with sexual health information may also be shaped by social norms and by the visibility and regulation of sexual content on digital platforms. SHISB is therefore both an individual information-seeking behavior and a socially situated practice.

As of December 2023, China boasts 1.092 billion Internet users, with an Internet penetration rate of 77.5%.^2^ The rapid expansion of the Internet, coupled with the proliferation of new media forms and applications, such as mobile devices, cloud computing, and social media, has made health information more accessible than ever before (9). Since its inception, social media has attracted a substantial user base. By September 2023, data from Quest Mobile indicated that there were 1.088 billion active users across five major new media platforms: Douyin, Kuaishou, XiaoHongshu, Bilibili, and Weibo. Social networks serve not only as platforms for interpersonal connection but also as rich resources for diverse forms of information. These platforms enable users to share and exchange information instantaneously, positioning them as a primary source of health-related content. In the era of mobile Internet, social media usage has been deeply ingrained into the daily lives of college students, largely due to the widespread use of smartphones (10). University students commonly use the Internet to seek health information, including information related to sexual health (11, 12). This reliance on online sources makes the quality and credibility of sexual health information an important concern.

Recent research indicates that the digitalization of sexual and reproductive health involves more than simply expanding access to information. Digital technologies can facilitate access to sexual and reproductive health information, education, and services, while also raising concerns about data governance, privacy, algorithmic transparency, equity, and accountability (13). Evidence from China further suggests that different forms of social media engagement may produce uneven learning outcomes. Zhang et al. (14) found that information elaboration was positively associated with both subjective and factual reproductive health knowledge, whereas public reposting was positively associated with subjective knowledge but negatively associated with factual knowledge, suggesting a possible illusion of knowing. Related research showed that sexual and reproductive health stigma and misinformation exposure were associated with information avoidance and misperceptions, with information overload intensifying some of these relationships (15). Pfender and Caplan (16) further examined sponsorship and medical-disclaimer cues in contraceptive messages communicated by social media influencers, highlighting the importance of source and message characteristics in digital sexual health communication. Taken together, these studies suggest that social media are not neutral or uniformly beneficial sources of sexual health information. Their value depends partly on how users evaluate the credibility, quality, relevance, and usefulness of the available content.

Against this background, the present study adopts the Comprehensive Model of Information Seeking (CMIS) to examine Chinese college students' sexual health information-seeking behavior (SHISB) on social media. Specifically, it tests whether perceived direct or vicarious experience, risk-oriented salience, beliefs about health management, and perceived information carrier characteristics are associated with perceived utility of sexual health information (SHI), and whether perceived information carrier characteristics and perceived utility of SHI are associated with SHISB. Because CMIS emphasizes individual-cognitive and information-evaluation processes, it is used here as a focused rather than comprehensive framework.

## 2 Literature review

### 2.1 CMIS and its theoretical boundaries

**Figure 1:**
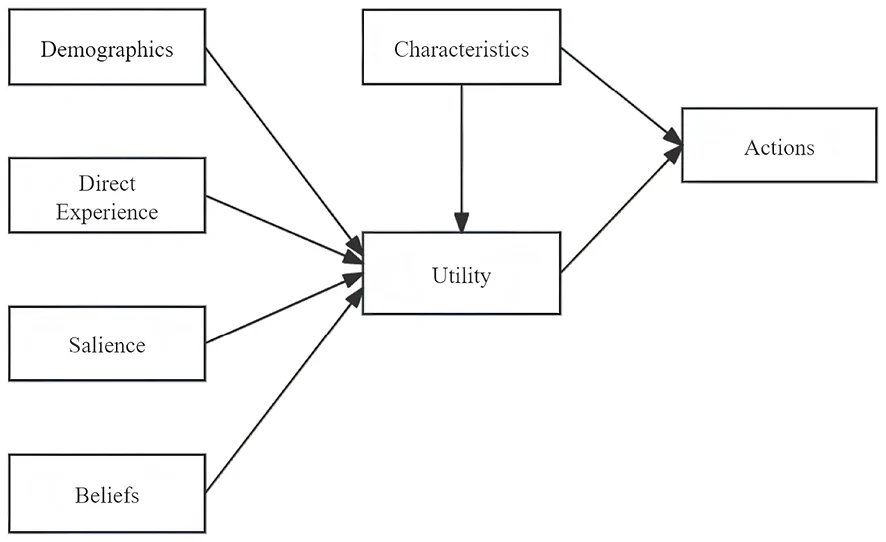
Comprehensive Model of Information Seeking.

The Comprehensive Model of Information Seeking (CMIS), proposed by Johnson and Meischke, integrates insights from the Health Belief Model, Uses and Gratifications theory, and media exposure and evaluation research (2). It identifies two broad sets of antecedents of health information seeking (as indicated in Figure 1): health-related factors, including demographic characteristics, direct experience, salience, and beliefs; and information-carrier factors, including source credibility, channel characteristics, perceived utility, and subsequent information-seeking actions. Originally developed to explain breast cancer-related information seeking through print media, CMIS has since been applied to a range of health contexts, including smoking-related information seeking, cancer information scanning, chronic illness information behavior, women's health information seeking, and online health information exchange (2, 17–20). These studies suggest that CMIS remains useful for examining how individuals evaluate information sources and how perceived utility is associated with health information-seeking behavior.

Recent health communication research, however, suggests that information seeking should not be treated as a purely individual or linear process. A meta-analysis of health information seeking has shown that this field draws on multiple theoretical traditions rather than one unified model, including risk information seeking, uncertainty management, planned risk information seeking, and health belief approaches (21). Similarly, Lewis et al. (22) distinguish active information seeking from less deliberate information scanning and show that information seeking is associated not only with issue relevance and risk perception but also with perceived social norms. These findings are important for the present study because sexual health information seeking on social media may involve deliberate search, incidental exposure, peer circulation, and platform recommendation at the same time. Therefore, CMIS provides a useful but partial framework: it helps explain the cognitive evaluation of information utility, but it cannot fully capture the broader communicative environment in which sexual health information becomes visible, acceptable, or avoidable.

The application of CMIS to sexual health information seeking also requires theoretical caution because sexual health is not merely the absence of disease, dysfunction, or risk. Sexual health information seeking cannot be understood only as a response to knowledge deficits or disease-related threats. Models that emphasize information need, perceived risk, and perceived utility may unintentionally reproduce an information-deficit logic, in which individuals are treated primarily as rational processors of neutral health information. This logic is valuable for modeling certain cognitive associations, but it may understate the cultural, moral, relational, and political conditions under which sexuality is discussed, avoided, or normalized.

This point is especially relevant in digital environments. Critical digital health scholarship argues that digital health technologies and platforms are not neutral channels through which health information simply flows; rather, they shape what counts as visible, credible, shareable, and actionable health knowledge (23, 24). Southerton et al. (25) demonstrated how content classification systems shape the sexual citizenship of lesbian, gay, bisexual, transgender, queer/questioning, and other sexual and gender minority communities (LGBTQ+) by regulating the visibility of sexual content. Are (26) similarly shows that sex educators, sexual health brands, and other sex-related accounts may be affected by content deletion, shadowbanning, and de-platforming on Instagram and TikTok. These studies suggest that students' sexual health information seeking is not shaped only by individual beliefs or perceived utility; it may also be shaped by platform moderation, algorithmic visibility, content regulation, and the moral classification of sexual topics.

Recent discussions of digital sexual health also move beyond the narrow model of information transmission. Albury and Hendry (27) argue that digital sexual health should be understood through information, influence, ritual, and participation, rather than simply through the delivery of accurate facts. This view is useful for the present study because Chinese college students may encounter sexual health content not only through formal educational materials, but also through influencer discourse, peer sharing, lifestyle content, platform recommendation, and everyday social media participation. In this sense, seeking sexual health information on social media may be simultaneously instrumental, relational, identity-related, and culturally negotiated. Connelly et al. (28) similarly show that electronic health (eHealth) literacy alone does not independently predict health decision-making quality; its role depends on users' motivation, ability to process information, and complexity of seeking strategies. This finding further cautions against assuming that providing information or improving access will automatically lead to better health understanding or behavior.

Social representations theory and rhetorical psychology further clarify why sexual health information seeking cannot be reduced to private cognition. Sexual-health meanings are collectively shaped through communication and may be anchored in privacy, shame, romance, modernity, personal responsibility, family expectations, and moral propriety (29–31). Students may therefore regard seeking SHI as responsible and useful while also viewing discussions of sex as private or socially risky, reflecting the ideological dilemmas described by Billig (32). From a social ecological perspective, these tensions are further shaped by peer norms, institutional contexts, and platform governance (33). These contextual influences are not tested as variables here; rather, they define the boundary within which the CMIS-based associations should be interpreted.

### 2.2 Demographic factors and perceived utility of SHI

Perceived utility of health information refers to individuals' evaluations of the usefulness of information in relation to their needs, goals, and problem-solving situations. In this study, it primarily captures students' instrumental evaluations of whether sexual health information (SHI) on social media is accessible, understandable, helpful, important, and capable of addressing their concerns. Previous studies have reported positive associations between perceived utility and health information-seeking behavior (34, 35). For example, the perceived utility of both sponsored and non-sponsored Internet sources predicts how frequently American adults seek information about prescription medications (36). Similarly, perceived utility has been shown to predict online information-seeking behavior related to mental health (20). Because individuals' evaluations of information utility may vary across demographic groups, the following discussion considers how demographic characteristics are associated with the perceived utility of health information.

According to Delorme et al. (36) demographic factors, such as gender, race, health insurance, level of education, and income, can influence how individuals seek and evaluate health-related information. Johnson and Meischke (2) reported that demographic factors are related to information utility. Research on undergraduates at Mzumbe University in Tanzania showed that women are more likely than men to seek sexual and reproductive health information (37). In addition, Zhang et al. (20) found that romantic relationship status played a role in shaping the health information-seeking behavior of Chinese women on Douyin. While the CMIS model has been successfully applied across different cultural contexts, some studies suggested that age, romantic relationship status, income, and race can affect the efficacy of the model (17, 38, 39). A study in Hong Kong found that the CMIS model was effective for explaining online health information-seeking behavior among certain generations but failed to explain the behavior of the Boomer generation (38). In the context of sexual health, this study included marital and romantic status as a factor to explore whether it impacts college students' SHISB. Gender and romantic relationship status are particularly relevant in sexual health contexts because they may be associated with perceived vulnerability, privacy concerns, interpersonal responsibility, and willingness to access sensitive information. Given the relatively homogeneous age distribution of the present college student sample, age was not examined as a focal demographic variable in this study. Studies have shown no association between age and risky sexual behaviors (40). In addition, because the study focused on free and widely used social media platforms, economic status was not included in the present model, although socioeconomic differences may still shape broader access to digital health resources. Gender and romantic relationship status were included as key variables that may significantly affect the perceived utility of health information. Sexual orientation and sexual identity may also shape sexual health information needs, perceived stigma, privacy concerns, and access barriers. These variables were not included in the present survey because of the sensitivity of the topic and the initial focus on testing a CMIS-based model, and this omission is addressed in the limitations section. Based on the above findings, the following hypotheses are proposed:

H1a: Perceived utility of SHI differs by gender.
H1b: Perceived utility of SHI differs by romantic relationship status.

### 2.3 Direct or vicarious experience and perceived utility of SHI

Direct experience is a critical factor in shaping health information-seeking behavior, particularly in terms of individuals' self-evaluated health status. Empirical studies have consistently demonstrated that direct experience with illness is associated with proactive information-seeking behavior (41). Furthermore, perceived health status may be related to media use for health information acquisition (42). In a study on Chinese women seeking mental health information on Douyin, Zhang et al. (20) found that direct experience can positively influence the perceived utility of mental health information. Moreover, direct experience affects the perceived utility of information through emotional symptoms and social networks (19). Research on prescription drug information-seeking behavior revealed that the utility of professional interpersonal channels is positively correlated with direct experience (36). Hartoonian et al. demonstrated that direct experience modifies the impact of cancer-related information on its utility. For college students, direct experience may include both personal experience and perceived vicarious experience involving friends or family members. However, because sexuality is often treated as a private and sensitive topic in the Chinese sociocultural context, students may not have accurate knowledge of family members' sexual health status. Therefore, family-related items should be interpreted as perceived vicarious experience rather than objective clinical information. These direct experiences can enhance the perceived relevance and usefulness of the information they seek (43). Consequently, the following study hypothesis is put forth:

H2: Perceived direct or vicarious experience with sexual health issues is positively associated with perceived utility of SHI.

### 2.4 Risk-oriented salience and perceived utility of SHI

Salience refers to the degree of importance or urgency attached to specific health information by an individual. In the present study, salience is operationalized primarily as risk-oriented salience, focusing on perceived seriousness, fear, bodily discomfort, and negative social consequences. This concept is directly linked to the perceived health risks by the individual. Salience provides the underlying motivation for information-seeking. Individuals who perceive a health issue as more serious or personally relevant may be more likely to evaluate related information sources as useful. In the original CMIS model, Johnson and Meischke (2) operationalized salience by examining an individual's perceived likelihood of developing cancer and their fear of the disease. They found that salience influences the perceived usefulness of information sources. Basnyat et al. (18) identified a negative correlation between salience and the utility of the Internet. The more severe an individual perceives a disease to be, the more they tend to view the Internet as insufficient or ineffective. Salience has been shown to be a predictor of the utility of information sources in a study on reproductive health information-seeking among young women in Peru (44). Hartoonian et al. defined cancer salience as an individual's perception of cancer as a health threat. They confirmed that cancer salience affects the selection of information sources and the scope of cancer-related information-seeking (43). Salience directly impacts the perceived utility of information carriers, as demonstrated in a study on smokers' health information-seeking behavior (19). Essentially, salience functions as a driving force for action, with individuals allocating more attention to information they perceive as important, relevant, or urgent. In the CMIS model, salience influences the perceived utility of information through cognitive and emotional pathways.

H3: Risk-oriented salience is positively associated with perceived utility of SHI.

### 2.5 Beliefs and perceived utility of SHI

Belief, in the context of health information-seeking behavior, refers to an individual's confidence in their ability to manage health-related issues through effective preventative, treatment, and control strategies, such as self-efficacy and response-efficacy (45). Self-efficacy reflects an individual's belief in their ability to manage their own health, while response efficacy involves the perception of the effectiveness of actions such as seeking information to address health-related concerns. In the present study, belief is interpreted not only as sexual-health-related self-efficacy and response efficacy, but also as a broader sense of responsible health self-management, because some items reflect students' general confidence in managing their health. The beliefs in the original information-seeking synthesis model are more closely aligned with response efficacy or people's perceptions of the efficacy of different cancer-related medical procedures. Johnson and Meischke (2) employed the Benefits and Drawbacks measure to investigate women's perceptions of the benefits and drawbacks of the breast cancer screening process. The investigation of self-efficacy and online health information seeking has shown that greater self-efficacy is linked to more frequent health information searches (46). Furthermore, Fung et al. (47) applied the information synthesis model in connecting Hong Kong citizens' health information seeking to chronic diseases, revealing that belief positively impacts the utility of information carriers. A study utilizing the information-seeking model was carried out (17), indicating that belief can impact effectiveness to some extent. In this study, self-efficacy refers to students' confidence in searching for, evaluating, and using online SHI, whereas response efficacy refers to their belief that seeking SHI on social media may help them understand or address sexual health concerns. Therefore, the following hypothesis is posited:

H4: Beliefs about sexual health management and information seeking are positively associated with perceived utility of SHI.

### 2.6 Information carrier characteristics, perceived utility of SHI, and SHISB

In 1993, Johnson and Meischke (2) defined information carrier characteristics as the attributes of the medium through which health information is disseminated, including the credibility, tone, and comprehension of the content. According to the complete model of information seeking, the attributes of an information carrier are associated with both information-seeking behavior and perceived utility. The operational definitions of information carrier characteristics vary across studies. One key attribute of an information carrier is the reliability of the information source (18). Variables such as information quality and ease of comprehension were measured (43). There is a relationship between individuals' online health information-seeking behavior and the reliability of the information encountered (48). This study centered on college students' sexual health issues and used an information credibility scale to assess information carrier characteristics. Moreover, in this study, information carrier characteristics refer to students' perceived accuracy, reliability, quality, completeness, objectivity, and timeliness of SHI on social media. These measures capture subjective evaluations of information quality rather than objective platform features. Therefore, they do not directly assess platform governance, algorithmic visibility, content moderation, or regulatory constraints, although these factors may also shape students' access to sexual health information. Based on the above, the research hypothesis is proposed:

H5a: Perceived information carrier characteristics are positively associated with perceived utility of SHI.
H5b: Perceived information carrier characteristics are positively associated with SHISB.

### 2.7 Perceived utility of SHI and SHISB

Beyond possible demographic differences in perceived utility, previous research has also examined whether perceived utility directly predicts information-seeking behavior. A study of cigarette smoking information revealed a positive correlation between perceived utility and information-seeking behavior (19). Fetherston (49) demonstrated that individuals' cognitive evaluation of the Internet's utility significantly and positively predicts their intention to seek online career-related information. According to a study on the health-seeking behavior of college students, perceived utility and perceived ease of use have been found to be positively associated with searching intention, which in turn positively affects their online health information-searching behavior (50). College students' health information seeking on social media platforms may be associated with their perception of utility (51). In online SHISB, perceived utility of SHI may influence students' use of social media, but it does not capture all relevant motivations, as students may still hesitate to seek information they consider useful because of privacy concerns, stigma, peer norms, or family and moral expectations. Therefore, the following hypotheses are proposed:

H6a: Perceived utility of SHI is positively associated with SHISB.
H6b: Perceived utility of SHI serves as an indirect statistical pathway linking perceived information carrier characteristics and SHISB.

## 3 Methods

### 3.1 Measures

Previous research has demonstrated that the comprehensive model of information-seeking (CMIS) possesses strong applicability and explanatory power in predicting and explaining individual information-seeking behavior (52). The model provides a foundational framework for examining the hypothesized associations among health-related factors, information-carrier factors, perceived utility, and information-seeking behavior. This study applied the CMIS to the context of sexual health to examine factors associated with college students' SHISB on social media platforms.

**Figure 2:**
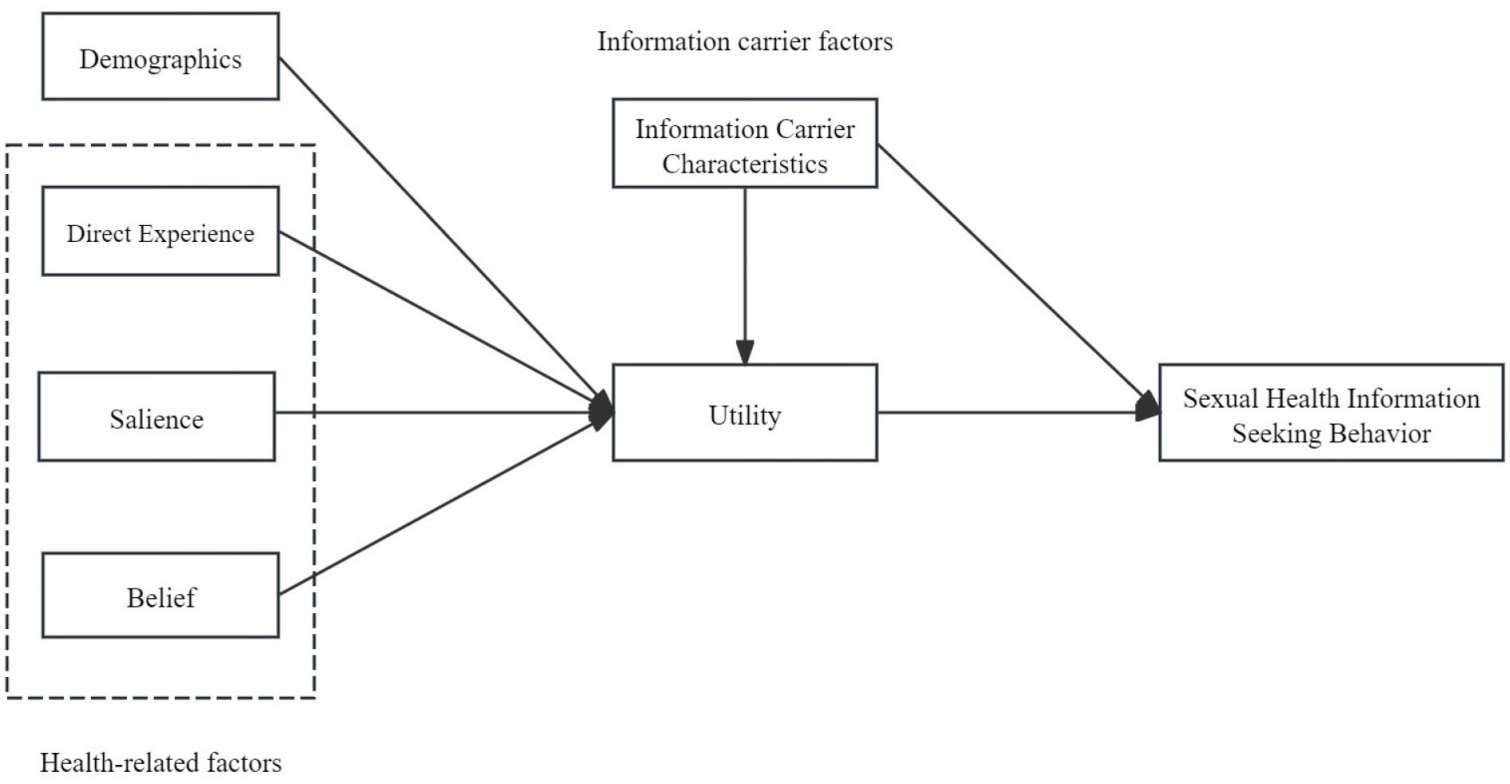
The CMIS model of this study.

Based on relevant literature, this study defined key factors within the CMIS framework, including direct experience, salience, belief, information carrier characteristics, information-seeking behavior, and utility. Based on these variables, hypotheses were formulated, and an explanatory model was constructed (as shown in Figure 2).

The questionnaire items were developed based on the operational definitions of CMIS constructs and adapted from prior studies on health information seeking and online health information behavior. Items for direct experience, salience, beliefs, information carrier characteristics, perceived utility, and SHISB were revised to fit the context of sexual health information seeking on Chinese social media platforms. Because the survey was administered in Chinese, the initial Chinese items were developed through a combination of translation, contextual adaptation, and discussion among the research team. Where items were adapted from English-language studies, bilingual members of the research team checked the conceptual equivalence between the English source items and the Chinese version. The translated items were back-translated by an independent bilingual researcher, and discrepancies were discussed and resolved by the research team. To improve content validity, the draft questionnaire was reviewed by two content experts, one specializing in health communication research and the other in sexual health education. The experts assessed whether the items were conceptually consistent with the CMIS constructs, whether the wording was suitable for Chinese college students, and whether sensitive sexual health items were understandable and ethically appropriate. Based on their feedback, ambiguous expressions were revised, several items were reworded to reduce potential discomfort, and the order of some questions was adjusted. The final questionnaire comprised four sections, (1) an introductory text description, (2) questions on social media use for SHISB among college students, addressing the aforementioned variables, (3) utilization of social media platforms (e.g., WeChat, Weibo, Douyin, Kuaishou, and Xiaohongshu), and (4) demographic data (e.g., gender, romantic relationship status). All items were scored using a five-point Likert scale (1 denoting strongly disagree and 5 representing strongly agree). The Likert scale was used to assess the attitudes, interests, and opinions of respondents. In order to ensure response validity, this study embedded attention-check items (e.g., participants were asked to select a specified response option for an attention-check item.) to identify inattentive respondents during data cleaning.

It should be noted that the operationalization of sexual health in this study has a risk-oriented emphasis. The measurement items did not fully cover the positive dimensions of sexual health. In particular, the direct experience items focused on whether respondents, their family members, or their friends had experienced sexual health problems, while the salience items focused on seriousness, the need for attention, negative effects on social life, bodily discomfort, and fear of sexual health problems or diseases. These items therefore mainly captured risk-oriented sexual health concerns rather than broader positive dimensions such as intimacy, sexual wellbeing, pleasure, relationship quality, or identity affirmation. However, this does not mean that all items were disease- or threat-oriented. The belief items measured students' confidence in health management, prevention, and the usefulness of seeking sexual health information. The information carrier items measured perceived accuracy, reliability, quality, completeness, objectivity, and timeliness, while the items measuring perceived utility of SHI assessed accessibility, helpfulness, importance, understandability, usefulness, and problem-solving value. Therefore, the findings should be interpreted as reflecting a CMIS-based model of risk-oriented sexual health concerns and instrumental evaluations of online sexual health information, rather than a comprehensive measurement of the full WHO conception of sexual health. Future research should develop culturally validated measures that include both risk-oriented and positive dimensions of sexual health.

### 3.2 Cognitive interview

Before the formal survey, cognitive interviews were conducted with 30 college students to assess item clarity, questionnaire length, response burden, and respondents' comfort with sensitive sexual health questions. Participants were invited to provide feedback on unclear wording, sensitive expressions, and the order of questions. Based on their feedback, several items were reworded to improve clarity and reduce potential discomfort, and the order of some items was adjusted. Brief cognitive feedback was collected from participants in cognitive interviews to check whether sensitive terms such as “sexual health issues" and “sexual health-related information" were understood as intended. The completion times in the cognitive interviews were also used to determine the response-time threshold for data cleaning. Based on the results of the cognitive interviews and the research team's assessment of the minimum time required to read and answer the questionnaire attentively, responses completed in less than 80 s were considered unlikely to reflect careful reading and were excluded.

### 3.3 Data collection

Data collection was conducted using Wenjuanxing, a widely adopted online survey platform in China. The survey link was initially shared through popular social media platforms (such as WeChat and Weibo), targeting university-affiliated user groups. The English and Chinese versions of the questionnaire are provided in Supplementary Tables S1, S2, respectively. Considering the sensitivity of sexual health topics in China, the questionnaire was anonymous and did not collect names, student identification numbers, phone numbers, Internet Protocol addresses, or other directly identifiable information. Participants were informed that participation was voluntary, that they could withdraw at any time, and that the data would be used only for academic research. Sensitive questions were phrased in general terms and participants were allowed to skip the survey before submission if they felt uncomfortable. In order to enhance sample diversity, a snowball sampling method was employed, encouraging participants to refer peers within their academic networks. Given the sensitivity of the topic and the high reliance of Chinese college students on social media, this approach helped reduce resistance to surveys on sexual health issues by leveraging established social networks. The survey was officially distributed on May 18, 2024, and remained open for 30 days, yielding 335 responses. Because all questionnaire items were set as required fields in Wenjuanxing, there were no item-level missing values in the submitted responses. Therefore, missing data imputation was not performed. Following standard data-cleaning procedures, invalid responses were excluded based on three criteria: (1) inconsistency with attention-check items, (2) systematic response patterns (e.g., >90% identical answers), and (3) completion times under 80 s, a threshold informed by cognitive interviews. Finally, 283 valid responses remained, reflecting an 84% validity rate.

### 3.4 Data analysis

Data were analyzed using the Statistical Package for the Social Sciences (SPSS), version 26.0 (IBM Corp., Armonk, NY, USA), and Analysis of Moment Structures (AMOS), version 22.0 (IBM Corp., Armonk, NY, USA). Descriptive statistics and correlation analyses were first conducted. Confirmatory factor analysis (CFA) was then used to assess the measurement model, and structural equation modeling (SEM) was used to examine the hypothesized associations among the constructs. Maximum likelihood estimation was used in AMOS. Following established guidelines for SEM (53), model fit was evaluated using the chi-square-to-degrees-of-freedom ratio (χ^2^/df), root mean square error of approximation (RMSEA), standardized root mean square residual (SRMR), comparative fit index (CFI), and Tucker–Lewis index (TLI). Values of χ^2^/df below 3.0, CFI and TLI values of 0.90 or higher, and RMSEA and SRMR values of 0.08 or lower were considered indicative of acceptable model fit, while RMSEA values of 0.06 or lower were interpreted as indicating close fit (54).

## 4 Results

### 4.1 Reliability and validity test

The reliability of the six constructs, namely direct experience, salience, beliefs, information carrier characteristics, perceived utility of SHI, and SHISB, was examined using Cronbach's alpha. As shown in Table 1, the Cronbach's alpha coefficients ranged from 0.81 to 0.93, all exceeding the recommended threshold of 0.70. Specifically, the reliability coefficients were 0.81 for direct experience, 0.90 for salience, 0.85 for beliefs, 0.93 for information carrier characteristics, 0.92 for perceived utility, and 0.89 for SHISB. These results indicate satisfactory internal consistency for all constructs.

**Table 1:** Reliability coefficients of variables.

Variables	Cronbach's *α*
1. Direct experience	0.81
2. Salience	0.90
3. Beliefs	0.85
4. Information carrier characteristics	0.93
5. Perceived utility of SHI	0.92
6. SHISB	0.89

Confirmatory factor analysis was then conducted to assess the construct validity of the measures. As shown in Table 2, the construct-level CFA models generally demonstrated adequate fit across most indices. The three-item direct or vicarious experience construct was just-identified (df = 0); therefore, its global fit indices were not informative. For the remaining constructs, the RMSEA values ranged from 0.05 to 0.16, the SRMR values ranged from 0.02 to 0.04, the TLI values ranged from 0.93 to 0.98, and the CFI values ranged from 0.95 to 0.99. The salience, beliefs, information carrier characteristics, and utility constructs showed adequate fit across most indices. For the SHISB construct, the SRMR (0.04), TLI (0.96), and CFI (0.96) indicated acceptable fit, although the RMSEA was relatively high (0.16). Given the very low degrees of freedom of this model (df = 2), the RMSEA value should be interpreted with caution. Taken together, the construct-level CFA results provided generally adequate support for the measurement model.

**Table 2:** Model fitting index of confirmatory factor analysis.

Variables	χ^2^	df	RMSEA	SRMR	TLI	CFI
1. Direct experience	0.00	0	–	–	–	–
2. Salience	66.47	37	0.06	0.02	0.97	0.98
3. Beliefs	21.93	12	0.05	0.02	0.93	0.95
4. Information carrier characteristics	26.95	11	0.06	0.02	0.98	0.99
5. Perceived utility of SHI	24.24	11	0.07	0.03	0.98	0.98
6. SHISB	17.35	2	0.16	0.04	0.96	0.96

Discriminant validity was further assessed in SPSS 26.0 using the Fornell-Larcker criterion. The inter-construct correlations were calculated based on the composite scores of the six constructs, and the square root of the average variance extracted (AVE) for each construct was placed on the diagonal of the correlation matrix. According to the Fornell-Larcker criterion, discriminant validity is supported when the square root of AVE for each construct exceeds its correlations with other constructs. As shown in Table 3, all diagonal values were higher than the corresponding off-diagonal correlations. The highest inter-construct correlation was observed between information carrier characteristics and perceived utility (*r* = 0.789). Although this correlation was relatively high, it remained slightly lower than the square roots of AVE for information carrier characteristics and perceived utility, which were 0.821 and 0.791, respectively. Therefore, the results supported acceptable discriminant validity.

**Table 3:** Discriminant (Fornell-Larcker criterion).

Construct	F1_DE	F2_SL	F3_BE	F4_MC	F5_MU	F6_SHISB
F1_DE	**0.767**					
F2_SL	0.206	**0.701**				
F3_BE	0.140	0.521	**0.704**			
F4_MC	0.082	0.261	0.437	**0.821**		
F5_MU	0.026	0.347	0.510	0.789	**0.791**	
F6_SHISB	−0.051	0.280	0.296	0.602	0.721	**0.820**

Bold diagonal values represent the square roots of AVE; off-diagonal values represent Pearson correlations among the composite construct scores.

The discriminant validity analysis was conducted using SPSS 26.0.

DE, direct experience; SL, salience; BE, beliefs; MC, information carrier characteristics; MU, perceived utility of SHI; SHISB, sexual health information-seeking behavior; AVE, average variance extracted.

Table 4 presents the scale-level descriptive statistics for the key variables, and item-level descriptive statistics are provided in Supplementary Table S3. Direct experience had a relatively low mean score (*M* = 1.89, SD = 0.32), suggesting that respondents reported limited personal or vicarious experience with sexual health problems. Salience had a relatively high mean score (*M* = 4.12, SD = 0.64), indicating that respondents generally regarded sexual health issues as important and serious. Beliefs also showed a relatively high mean score (*M* = 4.02, SD = 0.61), suggesting that respondents generally held positive beliefs about health management and sexual health information seeking. Information carrier characteristics had a moderate mean score (*M* = 3.43, SD = 0.70), indicating that respondents' evaluations of the accuracy, reliability, quality, completeness, objectivity, and timeliness of sexual health information on social media were moderate rather than highly positive. Perceived utility of SHI was above the midpoint of the scale (*M* = 3.73, SD = 0.62), showing that respondents generally recognized the usefulness and accessibility of social media for obtaining sexual health information. SHISB also showed a moderate to relatively high mean score (*M* = 3.69, SD = 0.74), suggesting that respondents had a certain level of engagement in sexual health information seeking on social media.

**Table 4:** Descriptive statistics of key variables (*N* = 283).

Variable	*M*	SD	Range
Direct experience	1.89	0.32	1.88–1.90
Salience	4.12	0.64	3.39–4.48
Beliefs	4.02	0.61	3.63–4.26
Information carrier characteristics	3.43	0.70	3.33–3.52
Perceived utility of SHI	3.73	0.62	3.60–3.84
SHISB	3.69	0.74	3.48–3.89

*M*, mean; SD, standard deviation. Range, minimum–maximum item mean within each scale.

### 4.2 Test of research hypotheses

Independent-samples *t*-tests and analysis of variance (ANOVA) were conducted to examine whether perceived utility of SHI differed by gender and relationship status. The results showed no statistically significant differences in perceived utility across gender or relationship status (*p*>0.05). Therefore, H1a and H1b were not supported. However, given the imbalanced gender distribution of the sample, with female respondents accounting for 73.5%, these null findings should be interpreted with caution.

**Figure 3:**
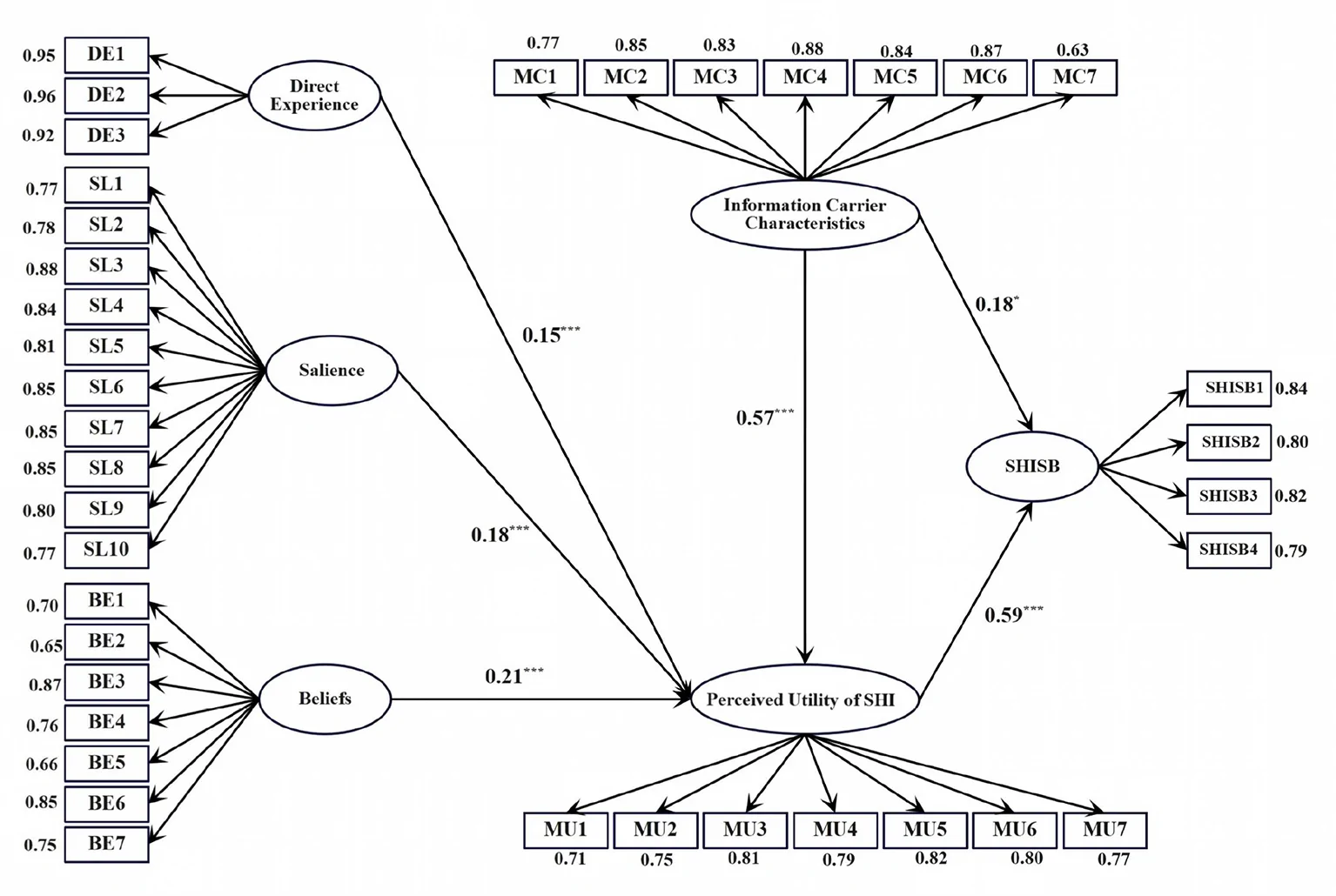
Final model with standardized path coefficients and factor loadings. ^*^ denotes *p* < 0.05, ^**^ denotes *p* < 0.01, ^***^ denotes *p* < 0.001.

To examine the proposed CMIS model, SEM analysis was conducted. The overall model fit was acceptable: χ^2^= 1,064.84, df = 552, *p* < 0.001; RMSEA = 0.054; SRMR = 0.05; TLI = 0.93; and CFI = 0.93. Although the chi-square test was significant, the other fit indices met the recommended criteria, indicating that the model adequately represented the relationships among the constructs. Figure 3 presents the standardized path coefficients and factor loadings of the final model. As shown in Figure 3, the standardized factor loadings were generally acceptable. Most factor loadings exceeded 0.70, while a few loadings were slightly below 0.70 but remained above 0.60. The lowest loading was observed for one item of information carrier characteristics, with a loading of 0.63. Given the satisfactory reliability coefficients reported in Table 1, the acceptable construct-level CFA fit indices reported in Table 2, and the standardized factor loadings shown in Figure 3, the measurement model was considered acceptable.

The SEM results showed that direct experience was positively associated with perceived utility (*β* = 0.15, *p* < 0.001), supporting H2. Salience also showed a significant positive association with perceived utility (*β* = 0.18, *p* < 0.001), supporting H3. Beliefs were positively associated with perceived utility (*β* = 0.21, *p* < 0.001), supporting H4. Information carrier characteristics was positively associated with perceived utility (*β* = 0.57, *p* < 0.001), supporting H5a. In addition, information carrier characteristics was positively associated with SHISB (*β* = 0.18, *p* < 0.05), supporting H5b. Perceived utility also was positively associated with SHISB (*β* = 0.59, *p* < 0.001), supporting H6a.

Among the antecedents of perceived utility of SHI, information carrier characteristics showed the strongest effect. This suggests that students' evaluations of the accuracy, reliability, quality, completeness, objectivity, and timeliness of sexual health information on social media played a particularly important role in shaping their perceived utility of social media for sexual health information seeking. In predicting SHISB, perceived utility showed a stronger effect than the direct effect of information carrier characteristics, indicating that students were more likely to seek sexual health information on social media when they perceived such platforms as useful, helpful, and easy to use.

To further test H6b, the mediating role of perceived utility of SHI in the relationship between information carrier characteristics and SHISB was examined using PROCESS Model 4 with 5,000 bootstrap samples. It should be noted that the coefficients reported in Table 5 are unstandardized coefficients from the PROCESS analysis, whereas the path coefficients reported in Figure 3 are standardized coefficients from the SEM analysis. As shown in Table 5, the total effect of information carrier characteristics on SHISB was significant [*B* = 0.598, SE = 0.051, 95% CI = (0.497, 0.699), *p* < 0.001]. After controlling for perceived utility, the direct effect of information carrier characteristics on SHISB remained significant [*B* = 0.186, SE = 0.071, 95% CI = (0.046, 0.325), *p* = 0.010]. The indirect effect through perceived utility was also significant [*B* = 0.412, SE = 0.061, 95% bootstrap CI = (0.296, 0.533)]. Because the confidence interval for the indirect effect did not include zero, H6b was supported. The mediation proportion was 69.0%, indicating that perceived utility accounted for a substantial proportion of the association between information carrier characteristics and SHISB. Since both the direct and indirect effects were significant, perceived utility partially mediated the relationship between information carrier characteristics and SHISB.

**Table 5:** Mediation analysis: indirect effect of information carrier characteristics on SHISB through perceived utility of SHI (PROCESS Model 4, *N* = 283).

Effect path	*B*	SE	95% Bootstrap CI	*p*-value
Total effect (MC → SHISB)	0.598	0.051	0.497, 0.699	< 0.001
Direct effect (MC → SHISB | Perceived utility of SHI)	0.186	0.071	0.046, 0.325	0.010
Indirect effect (MC → Perceived utility of SHI → SHISB)	0.412	0.061	0.296, 0.533	–
Mediation proportion	69.0%	–	–	–

Bootstrap samples = 5,000. All coefficients are unstandardized. CI, confidence interval.

The indirect effect was computed using percentile bootstrap confidence intervals.

The mediation proportion was calculated as the indirect effect divided by the total effect.

*B*, unstandardized coefficient; SE, standard error; CI, confidence interval; *M*, information carrier characteristics; SHISB, sexual health information-seeking behavior.

Overall, the results show that H2, H3, H4, H5a, H5b, H6a, and H6b were supported, whereas H1a and H1b were not supported. These findings provide empirical support for the applicability of the CMIS model in explaining Chinese college students' sexual health information-seeking behavior on social media.

## 5 Discussion

### 5.1 Principal findings and implications

First, in the present sample, perceived utility of SHI did not differ significantly by gender or romantic relationship status. This finding should be interpreted cautiously because the sample was gender-imbalanced and the male subgroup was relatively small. Therefore, the non-significant result should not be taken as evidence that gender or relationship status is irrelevant to SHISB. One possible interpretation is that the anonymity and accessibility of social media may reduce some barriers to evaluating sexual health information as useful, but future studies with gender-balanced and more diverse samples are needed.

Second, perceived direct and vicarious experience with sexual health issues was positively associated with perceived utility of SHI. This finding suggests that prior exposure to sexual health concerns may make related information more personally relevant and may increase students' evaluation of social media as a useful source of SHI. In the context of sexual health, direct experience should not be understood only as verified clinical experience. It may also include perceived experience derived from peers, family members, or everyday social interactions. Because sexuality is often treated as a private topic in the Chinese sociocultural context, students may not have accurate knowledge of family members' sexual health status. Therefore, family- and friend-related experience in this study should be interpreted as perceived vicarious experience rather than objective health information. Nevertheless, such perceived experience may still increase issue relevance and encourage students to pay closer attention to sexual health information on social media. This interpretation is generally consistent with CMIS research showing that direct experience is associated with perceived source utility and health information-seeking patterns (19, 20, 36, 41).

Third, risk-oriented salience was positively associated with perceived utility. Students who perceived sexual health concerns as more serious or threatening were more likely to regard social media as useful. Because salience was measured through threat-related items, however, this finding applies specifically to risk-oriented concerns and should not be generalized to positive motivations such as intimacy, curiosity, wellbeing, or identity affirmation. The result therefore supports CMIS within the present study's measurement boundaries rather than showing that sexual health information seeking is primarily threat-driven (27).

Fourth, beliefs were positively associated with perceived utility. In the CMIS framework, beliefs include self-efficacy and response efficacy, namely individuals' confidence in managing health-related issues and their belief that seeking information can help address such concerns. In this study, students with stronger beliefs about sexual health management and online information seeking tended to evaluate social media information as more useful. This may indicate that students who feel more capable of searching for, evaluating, and using SHI are more likely to perceive social media as a helpful information environment. At the same time, the belief construct should not be interpreted only as narrow sexual-health self-efficacy. Some belief items also reflect a broader orientation toward responsible health self-management. In the context of college students' social media use, confidence in managing health and seeking SHI may be linked to students' self-understanding as modern, health-literate, and responsible individuals. Therefore, beliefs may connect instrumental health management with identity-related motivations.

Finally, perceived information carrier characteristics were positively associated with both perceived utility and SHISB. Students who evaluated SHI on social media as more accurate, reliable, complete, objective, timely, and understandable also tended to report higher perceived utility and higher levels of SHISB. This finding is consistent with prior CMIS research emphasizing the role of information source evaluation in health information seeking (2, 18, 43). For sexual health topics, social media may provide a relatively private and accessible environment for seeking information, especially when formal sex education is insufficient or when students feel uncomfortable discussing sexuality face-to-face. However, social media should not be described as a stigma-free environment. Sexual health information on social media may still be shaped by stigma, moral judgement, peer visibility, platform regulation, and content moderation.

The information carrier characteristics measured here reflect students' subjective evaluations of information quality, not objective platform conditions. Because digital platforms are shaped by corporate and regulatory interests (23, 24), and sexuality-related content may be restricted through classification and flagging systems (25, 26), the positive association with SHISB should not be read as evidence that social media consistently provide high-quality sexual health information.

In addition, perceived utility of SHI served as an indirect statistical pathway linking perceived information carrier characteristics and SHISB. Because the data are cross-sectional, this association does not establish causal mediation. Moreover, perceived utility mainly captures instrumental evaluations of accessibility, understandability, helpfulness, and problem-solving value. Students may still hesitate or search selectively when information they regard as useful is also perceived as private or socially sensitive. Qualitative or mixed-method research is needed to examine how such tensions shape everyday information practices.

### 5.2 Limitations

This study has several limitations. First, the sample was obtained through convenience and snowball sampling, which may have introduced selection bias and limited representativeness. The data were collected mainly from college students in Guangdong through online channels. Therefore, the findings should not be generalized to all Chinese college students, especially those from inland, rural, or less digitally connected regions. China has substantial regional and urban-rural differences in digital literacy, sexual health education resources, and access to health services. Future studies should use multi-site, stratified, and mixed online-offline sampling strategies to improve external validity.

Second, the sample was gender-imbalanced, with female respondents accounting for 73.5% of the sample and the male subgroup including only approximately 75 participants. As a result, tests for gender differences may have been underpowered, increasing the risk of Type II error. The non-significant result for gender should therefore not be interpreted as evidence that gender is irrelevant to perceived utility or SHISB. Similarly, relationship-status differences should be interpreted cautiously because more than half of the respondents were single. Future research should recruit more balanced samples and examine whether gender, relationship status, and other demographic variables moderate sexual health information-seeking processes.

Third, the model and measures did not capture several potentially relevant dimensions, including perceived stigma, social risk, sexual orientation, sexual identity, and positive aspects of sexual health. Participants may also have interpreted the term “sexual health issues" differently, and family-related reports reflect perceived rather than verified experience. Future studies should develop culturally validated measures that encompass both risk-oriented and positive sexual health concerns.

Fourth, the cross-sectional design limits causal interpretation. Although the hypothesized paths were tested using SEM and an indirect statistical pathway was examined, the data cannot establish temporal order or causal mediation. The associations observed in this study should therefore be interpreted as statistical relationships rather than evidence of causal effects. Longitudinal, experimental, or mixed-method designs would be useful for examining how perceived utility, platform evaluation, and SHISB change over time.

Fifth, conducting questionnaire research on sexual health in China involves context-specific challenges. Even though the survey was anonymous, respondents may have underreported direct experience, avoided sensitive topics, or provided socially desirable answers because sexuality remains a private and morally sensitive issue in many family and educational settings. Although cognitive interviews were conducted to improve item clarity and sensitivity, the study did not include post-survey qualitative interviews or focus groups. Future studies could use these methods to better understand how students interpret sensitive items and negotiate shame, privacy, family expectations, and peer norms.

Finally, this study treated social media as a general information environment and did not fully distinguish among platform types, content formats, algorithmic recommendation mechanisms, or content moderation practices. Platforms such as Xiaohongshu, Bilibili, Douyin, Weibo, and WeChat may provide different affordances for anonymity, visibility, peer interaction, and professional health communication. In addition, data were collected in 2024, a post-coronavirus disease 2019 (COVID-19) period in which online health information seeking had become more normalized, while misinformation fatigue and skepticism toward online health content may also have increased. Future research should examine platform-specific differences, post-COVID temporal effects, and the role of platform governance in shaping sexual health information visibility and access.

## 6 Conclusion

This study examined sexual health information-seeking behavior (SHISB) on social media among Chinese college students through the Comprehensive Model of Information Seeking (CMIS). Based on a cross-sectional survey of 283 respondents, the results showed that perceived direct and vicarious experience, risk-oriented salience, beliefs, and perceived information carrier characteristics were positively associated with perceived utility. Perceived information carrier characteristics and perceived utility were also positively associated with SHISB, and perceived utility served as an indirect statistical pathway linking perceived information carrier characteristics and SHISB.

These findings suggest that CMIS is useful for examining individual-cognitive and information-carrier factors related to SHISB in the Chinese social media context. However, because the study mainly captured risk-oriented concerns and instrumental evaluations of information utility, CMIS should be understood as a focused rather than comprehensive framework for explaining sexual health information seeking.

For sexual health communication practice, the findings indicate that improving the perceived credibility, clarity, accessibility, and relevance of online sexual health information may help support students' information-seeking needs. However, because the study used a cross-sectional design and a convenience sample, the results should not be interpreted as evidence of causal effects. Future research should adopt longitudinal, mixed-method, and platform-specific designs to examine how students negotiate sexual health information seeking across different social media environments and sociocultural contexts.

## Data Availability

The raw data supporting the conclusions of this article will be made available by the authors, without undue reservation.

## Footnotes

1
https://www.who.int/health-topics/sexual-health#tab=tab_1

2
https://english.www.gov.cn/archive/statistics/202403/22/content_WS65fd41dac6d0868f4e8e5583.html
