# Supplementary material for: Determinants of sexual health information-seeking on social media among Chinese college students: an application of the Comprehensive Model of Information Seeking
Source: Front Public Health. 2026 Sep 1;14:1874571. doi: 10.3389/fpubh.2026.1874571 (PMC13574609; doi:10.3389/fpubh.2026.1874571)
Supplement: Supplementary file 1 [file Data_Sheet_1.pdf]

Supplementary Material

1 SUPPLEMENTARY TABLES

Table S1: The questionnaire used in this study in English

Q1: To what extent do you agree with these statements?					
	Strongly disagree	Disagree	Neutral	Agree	Strongly agree
I have or am suffering from sexual health issues.					
Members of my family have been or are struggling with sexual health issues.					
Friends of mine have been or are struggling with sexual health issues.					
Q2: To what extent do you agree with these statements?					
	Strongly disagree	Disagree	Neutral	Agree	Strongly agree
Having a sexual health problem is a serious matter.					
Suffering from sexual health diseases is a serious matter.					
Having sexual health problems needs to be taken seriously.					
Suffering from sexual health diseases needs to be taken seriously.					
Having sexual health problems will have a negative impact on my social life.					
Suffering from sexual health diseases will have a negative impact on my social life.					
Having sexual health problems will cause discomfort in my body.					
Suffering from sexual health diseases will cause discomfort in my body.					
I am very afraid of having sexual health problems.					
I am very afraid of suffering from sexual health diseases.					
Q3: To what extent do you agree with these statements?					
	Strongly disagree	Disagree	Neutral	Agree	Strongly agree
I can manage my own health.					
I have made some plans to improve my health.					

I can take good precautions to avoid being infected with “sexual health”-related diseases.					
Searching for sexual health-related information on social platforms can help me have a deeper understanding of sexual health.					
Searching for sexual health-related information on social platforms can help me treat sexual health diseases.					
I am confident that I can protect my sexual health.					
If I have sexual health-related problems, I am confident that I can solve them.					
Q4: To what extent do you agree with these statements?					
	Strongly disagree	Disagree	Neutral	Agree	Strongly agree
The information I obtain when searching for sexual health-related information on social media is accurate.					
The information I obtain when searching for sexual health-related information on social media is in line with reality.					
The information I obtain when searching for sexual health-related information on social media is true and reliable.					
The information I obtain when searching for sexual health-related information on social media is of high quality.					
The information I obtain when searching for sexual health-related information on social media is complete.					
The information I obtain when searching for sexual health-related information on social media is objective.					
The information I obtain when searching for sexual health-related information on social media is updated in a timely manner.					
Q5: To what extent do you agree with these statements?					
	Strongly disagree	Disagree	Neutral	Agree	Strongly agree
I can easily search for sexual health-related information on social platforms.					
I can find the information I need on social media in a short time.					

The information I obtain when searching for sexual health-related information on social media is important to me.					
The information I obtain when searching for sexual health-related information on social media is helpful to me.					
The information I obtain when searching for sexual health-related information on social media is easy to understand.					
The information I obtain when searching for sexual health-related information on social media is useful.					
The information I obtain when searching for sexual health-related information on social media can solve problems.					
Q6: To what extent do you agree with these statements?					
	Strongly disagree	Disagree	Neutral	Agree	Strongly agree
I search for sexual health-related information on social media platforms.					
I search for sexual health-related information on social media platforms when needed.					
I spend time searching for sexual health-related information on social media platforms.					
I also regularly search for sexual health-related information on social media platforms.					
Q7: In the past month, how often have you used each of the following social media platforms to seek information related to “sexual health”?					
	Never	Rarely	Sometimes	Often	Always
WeChat					
Weibo					
Douyin					
Kuaishou					
Xiaohongshu					
Zhihu					
Bilibili					
Other					
Q8: What is your gender?					
Male					
Female					
Q9: What is your current relationship status?					
Never been in a romantic relationship					

Previously in a romantic relationship but currently single	
Currently in a romantic relationship	
Married	

Table S2: 本研究的问卷

Q1:您在多大程度上同意这些观点?					
	非常不同意	不同意	中立	同意	非常同意
我曾经或现在有性健康问题的困扰					
我的家人曾经或现在有性健康问题的困扰					
我的朋友曾经或现在有性健康问题的困扰					
Q2:您在多大程度上同意这些观点?					
	非常不同意	不同意	中立	同意	非常同意
出现性健康问题是一件严重的事情					
患性健康疾病是一件严重的事情					
出现性健康问题是需要引起重视的					
患性健康疾病是需要引起重视的					
出现性健康问题会对我的社会生活产生不良影响					
患性健康疾病会对我的社会生活产生不良影响					
出现性健康问题会使我的身体出现不适					
患性健康疾病会使我的身体出现不适					
我很害怕自己出现性健康问题					
我很害怕自己患性健康疾病					
Q3:您在多大程度上同意这些观点?					
	非常不同意	不同意	中立	同意	非常同意
我能管理好自己的健康					
我已经制定了一些计划来改善自己的健康					
我可以做好防护以避免自己感染"性健康"相关疾病					
在社交平台上搜索性健康相关信息,可以帮助我深入了解性健康					
在社交平台上搜索性健康相关信息,可以帮助我治疗性健康疾病;					
我有信心能够守护我的性健康					

如果我出现了性健康相关问题，我有信心能够解决					
Q4:您在多大程度上同意这些观点?					
	非常不同意	不同意	中立	同意	非常同意
我在社交媒体搜索性健康相关信息，获得的信息是准确的					
我在社交媒体搜索性健康相关信息，获得的信息是符合现实（事实）的					
我在社交媒体搜索性健康相关信息，获得的信息是真实可信的；					
我在社交媒体搜索性健康相关信息，获得的信息质量很高；					
我在社交媒体搜索性健康相关信息，获得的信息是完整的；					
我在社交媒体搜索性健康相关信息，获得的信息是客观的；					
我在社交媒体搜索性健康相关信息，获得的信息是及时更新的；					
Q5:您在多大程度上同意这些观点?					
	非常不同意	不同意	中立	同意	非常同意
我在社交平台上很容易搜索到有关性健康相关信息。					
我能在短时间内在社交媒体中找到我需要的信息					
我在社交媒体搜索性健康相关信息，获得的信息对我是重要的；					
我在社交媒体搜索性健康相关信息，获得的信息对我是有帮助的。					
我在社交媒体搜索性健康相关信息，获得的信息是容易理解的					
我在社交媒体搜索性健康相关信息，获得的信息是有用的					
我在社交媒体搜索性健康相关信息，获得的信息是能解决问题的					
Q6:您在多大程度上同意这些观点?					
	非常不同意	不同意	中立	同意	非常同意
我会在社交媒体平台搜寻性健康相关信息					
我在有需要的时候会在社交媒体平台搜寻性健康相关信息					
我会花时间在社交媒体平台搜寻性健康相关信息					
我平常也会在社交媒体平台搜寻性健康相关信息					
Q7：在过去的1个月里，您使用下列社交媒体平台寻求“性健康”相关信息的频率如何?					

	从不	较少	有一些	较多	总是
微信					
微博					
抖音					
快手					
小红书					
知乎					
哔哩哔哩					
其他					
Q8: 您的性别:					
男					
女					
Q9：您的婚恋情况					
一直单身					
恋爱后分手					
恋爱中					
已婚					

Table S3: Item-level Descriptive Statistics of Measurement Items

Variable	Item	Mean	Median	Mode	Standard Deviation
Direct Experience	I have or am suffering from sexual health issues.	1.88	2	2	0.326
	Members of my family have been or are struggling with sexual health issues.	1.89	2	2	0.317
	Friends of mine have been or are struggling with sexual health issues.	1.9	2	2	0.304
Salience	Having a sexual health problem is a serious matter.	3.39	4	4	0.996
	Suffering from sexual health diseases is a serious matter.	4	4	4	0.94
	Having sexual health problems needs to be taken seriously.	4.43	5	5	0.693
	Suffering from sexual health diseases needs to be taken seriously.	4.48	5	5	0.659
	Having sexual health problems will have a negative impact on my social life.	3.94	4	4	0.932
	Suffering from sexual health diseases will have a negative impact on my social life.	4.01	4	4	0.926
	Having sexual health problems will cause discomfort in my body.	4.27	4	4	0.646

Table S3 continued from previous page

Variable	Item	Mean	Median	Mode	Standard Deviation
	Suffering from sexual health diseases will cause discomfort in my body.	4.34	4	4	0.646
	I am very afraid of having sexual health problems.	4.14	4	4	0.727
	I am very afraid of suffering from sexual health diseases.	4.24	4	4	0.72
Beliefs	I can manage my own health.	4	4	4	0.799
	I have made some plans to improve my health.	3.63	4	4	0.938
	I can take good precautions to avoid being infected with “sexual health”-related diseases.	4.26	4	4	0.675
	Searching for sexual health-related information on social platforms can help me have a deeper understanding of sexual health.	4.25	4	4	0.645
	Searching for sexual health-related information on social platforms can help me treat sexual health diseases.	3.84	4	4	0.939
	I am confident that I can protect my sexual health.	4.24	4	4	0.639
	If I have sexual health-related problems, I am confident that I can solve them.	3.98	4	4	0.746
Information Carrier Characteristics	The information I obtain when searching for sexual health-related information on social media is accurate.	3.51	3	3	0.852
	The information I obtain when searching for sexual health-related information on social media is in line with reality.	3.52	3	3	0.809
	The information I obtain when searching for sexual health-related information on social media is true and reliable.	3.46	3	3	0.817
	The information I obtain when searching for sexual health-related information on social media is of high quality.	3.36	3	3	0.869
	The information I obtain when searching for sexual health-related information on social media is complete.	3.33	3	3	0.856
	The information I obtain when searching for sexual health-related information on social media is objective.	3.45	3	3	0.795
	The information I obtain when searching for sexual health-related information on social media is updated in a timely manner.	3.47	3	3	0.839
Perceived Utility of SHI	I can easily search for sexual health-related information on social platforms.	3.73	4	4	0.837

Table S3 continued from previous page

Variable	Item	Mean	Median	Mode	Standard Deviation
	I can find the information I need on social media in a short time.	3.66	4	4	0.82
	The information I obtain when searching for sexual health-related information on social media is important to me.	3.77	4	4	0.747
	The information I obtain when searching for sexual health-related information on social media is helpful to me.	3.81	4	4	0.7
	The information I obtain when searching for sexual health-related information on social media is easy to understand.	3.84	4	4	0.694
	The information I obtain when searching for sexual health-related information on social media is useful.	3.75	4	4	0.719
	The information I obtain when searching for sexual health-related information on social media can solve problems.	3.6	4	4	0.812
SHISB	I search for sexual health-related information on social media platforms.	3.75	4	4	0.816
	I search for sexual health-related information on social media platforms when needed.	3.89	4	4	0.733
	I spend time searching for sexual health-related information on social media platforms.	3.64	4	4	0.878
	I also regularly search for sexual health-related information on social media platforms.	3.48	4	4	0.972
